# A basic model for assessing primary health care electronic medical record data quality

**DOI:** 10.1186/s12911-019-0740-0

**Published:** 2019-02-12

**Authors:** Amanda L. Terry, Moira Stewart, Sonny Cejic, J. Neil Marshall, Simon de Lusignan, Bert M. Chesworth, Vijaya Chevendra, Heather Maddocks, Joshua Shadd, Fred Burge, Amardeep Thind

**Affiliations:** 1 Department of Family Medicine, Department of Epidemiology & Biostatistics, Schulich Interfaculty Program in Public Health, Schulich School of Medicine & Dentistry, The University of Western Ontario, 1151 Richmond Street, London, Ontario N6A 3K7 Canada; 2 Department of Family Medicine, Department of Epidemiology & Biostatistics, Schulich School of Medicine & Dentistry, The University of Western Ontario, 1151 Richmond Street, London, Ontario N6A 3K7 Canada; 3 Department of Clinical and Experimental Medicine, University of Surrey, Guildford, Surrey GU2 7XH UK; 4 School of Physical Therapy, Faculty of Health Sciences, Department of Epidemiology & Biostatistics, Schulich School of Medicine & Dentistry, The University of Western Ontario, 1151 Richmond Street, London, Ontario N6A 3K7 Canada; 5 Science and Software Educator and Consultant, 58 Moraine Walk, London, Ontario N6G 4Y8 Canada; 6 Department of Family Medicine, Schulich School of Medicine & Dentistry, The University of Western Ontario, 1151 Richmond Street, London, Ontario N6A 3K7 Canada; 7 Department of Family Medicine, McMaster University, 100 Main Street West, 6th Floor, Hamilton, Ontario L8P 1H6 Canada; 8 Department of Family Medicine, Dalhousie University, 5909 Veterans Memorial Lane, Abbie J Lane Building, Room 8101B, Halifax, Nova Scotia B3H 2E2 Canada; 9 Department of Family Medicine, Department of Epidemiology & Biostatistics, Schulich Interfaculty Program in Public Health, Schulich School of Medicine and Dentistry, The University of Western Ontario, 1151 Richmond Street, London, Ontario N6A 3K7 Canada

**Keywords:** Primary health care, Computerized medical records, Electronic medical records, Data quality

## Abstract

**Background:**

The increased use of electronic medical records (EMRs) in Canadian primary health care practice has resulted in an expansion of the availability of EMR data. Potential users of these data need to understand their quality in relation to the uses to which they are applied. Herein, we propose a basic model for assessing primary health care EMR data quality, comprising a set of data quality measures within four domains. We describe the process of developing and testing this set of measures, share the results of applying these measures in three EMR-derived datasets, and discuss what this reveals about the measures and EMR data quality. The model is offered as a starting point from which data users can refine their own approach, based on their own needs.

**Methods:**

Using an iterative process, measures of EMR data quality were created within four domains: comparability; completeness; correctness; and currency. We used a series of process steps to develop the measures. The measures were then operationalized, and tested within three datasets created from different EMR software products.

**Results:**

A set of eleven final measures were created. We were not able to calculate results for several measures in one dataset because of the way the data were collected in that specific EMR. Overall, we found variability in the results of testing the measures (e.g. sensitivity values were highest for diabetes, and lowest for obesity), among datasets (e.g. recording of height), and by patient age and sex (e.g. recording of blood pressure, height and weight).

**Conclusions:**

This paper proposes a basic model for assessing primary health care EMR data quality. We developed and tested multiple measures of data quality, within four domains, in three different EMR-derived primary health care datasets. The results of testing these measures indicated that not all measures could be utilized in all datasets, and illustrated variability in data quality. This is one step forward in creating a standard set of measures of data quality. Nonetheless, each project has unique challenges, and therefore requires its own data quality assessment before proceeding.

**Electronic supplementary material:**

The online version of this article (10.1186/s12911-019-0740-0) contains supplementary material, which is available to authorized users.

## Background

The increased use of electronic medical records (EMRs) in Canadian primary health care practice [1–3] has resulted in an expansion of the availability of EMR data. These data are being put to uses such as quality improvement activities related to patient care, and secondary purposes such as research and disease surveillance [4, 5]. This has shifted the traditional use of medical records as an *aide-memoire* to that of a data collection system [6]. Yet the nature of the data that a primary health care practitioner requires for the care of patients can differ from what is needed for other purposes, for example, research [7]. Therefore, the overall assessment of the quality of these data can vary depending on their intended use. This characteristic of data quality is aligned with the concept of “fitness for purpose”, i.e. are the data of appropriate quality for the use to which they are going to be applied [8, 9].

Electronic medical records contain data that do not exist elsewhere, and can inform questions about primary health care; these data offer a unique window into patient care. As the foundation of the health care system, primary health care is where the majority of patient care is provided, and thus is a significant part of the system for which to consider data quality [10, 11]. Stakeholders interested in primary health care EMR adoption and use in Canada have recognized the importance of understanding data quality [12]. Current information regarding Canadian primary health care EMR data suggests there is variability in levels of quality. In particular, issues have been identified in the completeness of risk factor information [13, 14] chronic disease documentation [15], recording of weight and family history [14], and socio-demographic data quality [16] . This echoes the evidence from other countries [17–19], from studies conducted in the past [20–22] and in other health care settings [23]. Overall, these results reinforce that EMR data quality is an ongoing issue, particularly for researchers.

It is incumbent upon us therefore, as potential users of primary health care EMR data, to understand their quality in relation to the uses to which they are applied. For example, primary health care practitioners require tools that use EMR data to support the increasingly complex care of their patients [24]. Additionally, high quality data are needed for reporting on quality of care provision [25]. Decision support functions of the EMR work best when the system contains accurate information [26]. Researchers need data of high quality to reduce bias and the risk of erroneous conclusions in their studies. Decision-makers also seek standardized, aggregated PHC data (across EMRs) for policy-making and planning.

Tests of data quality, when defined in terms of fitness for purpose, thus vary across these three perspectives: clinical, research, and decision-making. Having measures in place with which to assess EMR data quality is a precursor to any assessment activity, and needed to underpin all three perspectives. While some guidance exists regarding data quality evaluation (please see Additional file 1: Appendix A), much of the recent primary health care EMR data quality literature focuses on either process steps [27], or the results of data quality assessments in one domain, such as completeness [13–15, 17]. In addition, there currently is no consensus on how data quality assessments should be approached, nor the measures of data quality that should be used [8].

In the following, we describe a process of conceptualizing, developing, and testing a set of measures of primary health care EMR data quality, within four domains: comparability; completeness; correctness; and currency. We share the results of applying these measures in three EMR-derived datasets, and discuss what this reveals about the measures and EMR data quality. This builds on previous EMR data quality work (see above and Additional file 1: Appendix A), but differs because we developed and tested multiple measures of data quality, within four domains, in three different EMR-derived primary health care datasets.

Herein we propose a basic model for assessing primary health care EMR data quality, comprising a set of data quality measures within four domains. This model is offered as a starting point from which data users can refine their own approach, based on their own needs.

## Methods

### Basic model of primary health care EMR data quality

Four overall tasks were completed in developing the basic model of primary health care EMR data quality: 1) conceptualizing data quality domains; 2) developing data quality measures; 3) operationalizing the data quality measures; and 4) testing the data quality measures.

#### Conceptualizing data quality domains

Focusing on the assessment of EMR data quality from the research perspective, we conceptualized the measurement of EMR data quality within four domains. The first is **comparability** which is aligned with the concept of reliability [28]. In the context of EMR data quality we can extend this concept to mean the degree to which EMR data are consistent with, or comparable to, an external data source [29, 30]; results of this comparison affect the generalizability of our analyses. Second, is **completeness** which is referred to by Hogan and Wagner as “..the proportion of observations made about the world that were recorded in the CPR [computer-based patient records]..” [31]. Third, **correctness** has been defined as “..the proportion of CPR observations that are a correct representation of the true state of the world..” [31]. This dimension is reflective of the concept of validity, i.e. “..the degree to which a measurement measures what it purports to measure” [28]. Finally, the fourth domain is **currency** or **timeliness** [32, 33] - the latter asks, “Is an element in the EHR [electronic health record] a relevant representation of the patient state at a given point in time?” [33]. We used a series of process steps to develop and test a set of EMR data quality measures, (defined as metrics or indicators of data quality) within these domains.

#### Developing the data quality measures

In the development phase, the research team conducted a literature review to identify measures of EMR data quality that had been used previously, as well as developing de novo measures. We were interested in creating measures that could be tested using structured EMR data, that were applicable across multiple EMRs, that were readily applied using the data within the EMR itself, and that addressed the four domains of comparability, completeness, correctness, and currency. Thus, through an iterative process of assessing the benefits and drawbacks of each potential measure according to these criteria, we created an initial set of measures.

#### Operationalizing the data quality measures

We conducted three steps to operationalize the measures. First, we identified test conditions to be used with the measures. The research team generated a list of thirteen conditions based on their prevalence in primary health care practice, previous use in EMR data quality research, and clinician team member input. After a process of assessment regarding the clinical importance of the conditions, the availability of relevant data in the EMR (i.e. would the condition be recorded in the cumulative patient profile or the problem list), and the feasibility of finding the data (i.e. presence of data in the structured portion of the EMR data vs. notes portion of the record), six conditions were selected for use: diabetes, hypertension, hypothyroidism, asthma, obesity, and urinary tract infection. Second, we needed to create case definitions so that patients with the test conditions could be identified (see Additional file 2: Appendix B). We could not use existing validated EMR case definitions that contain a billing code [34] because for two of the measures we needed to compare the proportion of patients who actually had diabetes and hypertension (according to our definition) against the proportion with a billing code for these conditions. Three family physician members of the team (SC, JNM, JS) assessed the case definitions that were created according to expected patient treatment practices and recording patterns in the EMR. Information including the problem list, medications, laboratory results, blood pressure readings, and BMI data contained in the databases was used. Multiple steps were undertaken to process each EMR data element used in the definitions. For example, free text recording of medication names and problem list entries were screened and verified by the clinical research team members. Third, we determined the specific details of each measure, for example the age ranges of the patients as applicable. Finally the statistical tests for the appropriate measures were determined. Please see Table 1 for details.

**Table 1:** Data Quality Measures

Domain	Measure	Operationalization of Measure
Comparability	Comparison of Database Population to a Standard Population	∙ Compare age-sex structure of the database population to an external/standard population (e.g. population census) using 5 year age bands, graph results ∘ Statistical test: Chi Square by patient age and sex ∙ Compute the mean and median age by sex for the database population and compare to an external/standard population
	Concordance of Test Conditions	∙ Compute crude and age standardized prevalence of test conditions (diabetes mellitus, hypothyroidism, hypertension, asthma, urinary tract infection, and obesity) within the database population, compare to published prevalence figures for the test conditions
Completeness	Sensitivity	∙ Calculate sensitivity values for test conditions of diabetes mellitus, hypertension, hypothyroidism, asthma, obesity and urinary tract infection. Use test condition definitions as the gold standard and billing code as the comparison standard.
	“Consistency of Capture” [46]	∙ Calculate: ∙ Percentage of all patients with 1 or more entries on problem list ∙ Percentage of all patients with 1 or more entries on allergy record, including “no allergies” ∙ Percentage of patients visiting in the last year of the database with 1 or more prescribed medications
	Recording of Blood Pressure, Height, and Weight	∙ Calculate proportion of patients with: ∙ 1 or more blood pressure recordings for patients 18 + years, males and females ∙ 1 or more height recordings for patients of all ages, males and females ∙ 1 or more weight recording for patients of all ages, males and females ∙ Statistical test: Chi Square by patient age and sex
	Recording of Blood Pressure among Patients Requiring a Blood Pressure Measurement	∙ Calculate: ∙ Percentage of patients with diabetes mellitus, with 1 or more blood pressure recordings within 12 months of date of onset of diabetes ∙ Percentage of patients with hypertension medications (2 or more oral anti-hypertensives, or 1 or more diuretics) with 1 or more blood pressure recordings within 12 months of date of onset of hypertension
Correctness	Positive Predictive Value	∙ Calculate positive predictive values (using same approach as for sensitivity) for test conditions of diabetes mellitus, hypertension, hypothyroidism, asthma, obesity and urinary tract infection
	Unlikely Combinations of Age & Specific Procedures	∙ Calculate the percentage of patients 10 or more years of age with a tetanus toxoid conjugate vaccine (diphtheria, haemophilus B, pertussis, polio, and tetanus) (which is usually reserved for children < 10 years of age)
Currency	Timeliness of Weight Recording for Patients with Obesity	∙ Calculate the percentage of obese patients with 1 or more weight recordings within 1 year of last visit in recorded in the database
	Timeliness of Visit for Pregnancy	∙ Calculate percentage of patients with a positive pregnancy laboratory test result and 1 or more visits within two months of the result
	Timeliness of Blood Pressure, Height, and Weight Recording	Calculate proportion of patients with values recorded no more than one year prior to their last visit in the database for: ∙ 1 or more blood pressure recordings for patients 18 + years, males and females ∙ 1 or more height recordings for patients of all ages, males and females ∙ 1 or more weight recording for patients of all ages, males and females Statistical test: Chi Square by patient age and sex

#### Testing of the data quality measures

Next we tested the measures sequentially in three datasets built from data extracted from three different EMR software products (herein referred to as dataset A, B, and C). The details of the datasets are as follows: dataset A - 43 family physicians from 13 sites contributed data for 31, 000 patients from Jan 1, 2006 to Dec 31, 2015; dataset B - 15 family physicians contributed data for 2472 patients from July 1st, 2010 to June 30, 2014; dataset C - 10 family physicians from 1 site contributed data for 14,396 patients from March 1st, 2006 to June 30, 2010 (please see Table 2). These datasets were created for the Deliver Primary Healthcare Information (DELPHI) project; this study is part of the DELPHI project. De-identified data are extracted from primary health care practices in Southwestern Ontario, Canada and combined to create the datasets which form the DELPHI database.

**Table 2:** Patient Characteristics in Each Dataset

	Dataset A		Dataset B		Dataset C	
Years of Data	January 1, 2006-December 31, 2015		July 1, 2010-June 30, 2014		March 1, 2006-June 30, 2010	
	#	%	#	%	#	%
Sex						
Males	14,619	47.2%	1126	45.6%	6614	45.9%
Females	16,381	52.8%	1346	54.4%	7782	54.1%
Total	31,000	100.0%	2472	100.0%	14,396	100.0%
Missing Sex	5		0		0	
Age	As of January 1, 2006		As of July 1, 2010		As of March 1, 2006	
Mean	39.4		48.7		38.4	
Median	41		53		38	
Missing Age	1367		0		5	

Note: cases with complete sex and age information are included

The datasets included in the DELPHI database are extracted from the EMR as a set of relational tables. For example, there is one table to store patient sex and age, and another table to store their scheduled appointments - these are linked by a unique patient identifier. The structure of the tables depends on the EMR software provider. For example, some EMRs provide discrete fields to enter height or weight information and specify the metric to be used, and drop down menus to select diagnosis codes. Other EMRs provide open fields for the provider to enter free text. Each dataset was analyzed separately to identify the location of the fields used in the data quality assessment. Datasets A and B had a higher proportion of structured fields for data entry, while Dataset C had several areas of free text that were searched and coded for analysis.

Written consent was obtained from all physician participants in the DELPHI project. The physicians are the data custodians of the patient’s EMR. DELPHI data extraction procedures, consent processes, and methods are described more fully elsewhere [35]. The DELPHI project was approved by The University of Western Ontario’s Health Sciences Research Ethics Board (number 11151E).

Within the process of testing the measures, several from the initial set were modified, or dropped, while others were added through the course of the study (e.g. % of patients with one or more entries on the problem list). We could not calculate several measures in dataset C (due to absence of laboratory values in a specific format for diabetes, and the different format of the problem list). However, we were able to calculate the remainder of the measures in the three datasets. This resulted in a final set of eleven measures (see Table 1).

## Results

### Data quality assessment

#### Comparability

**Fig. 1:**
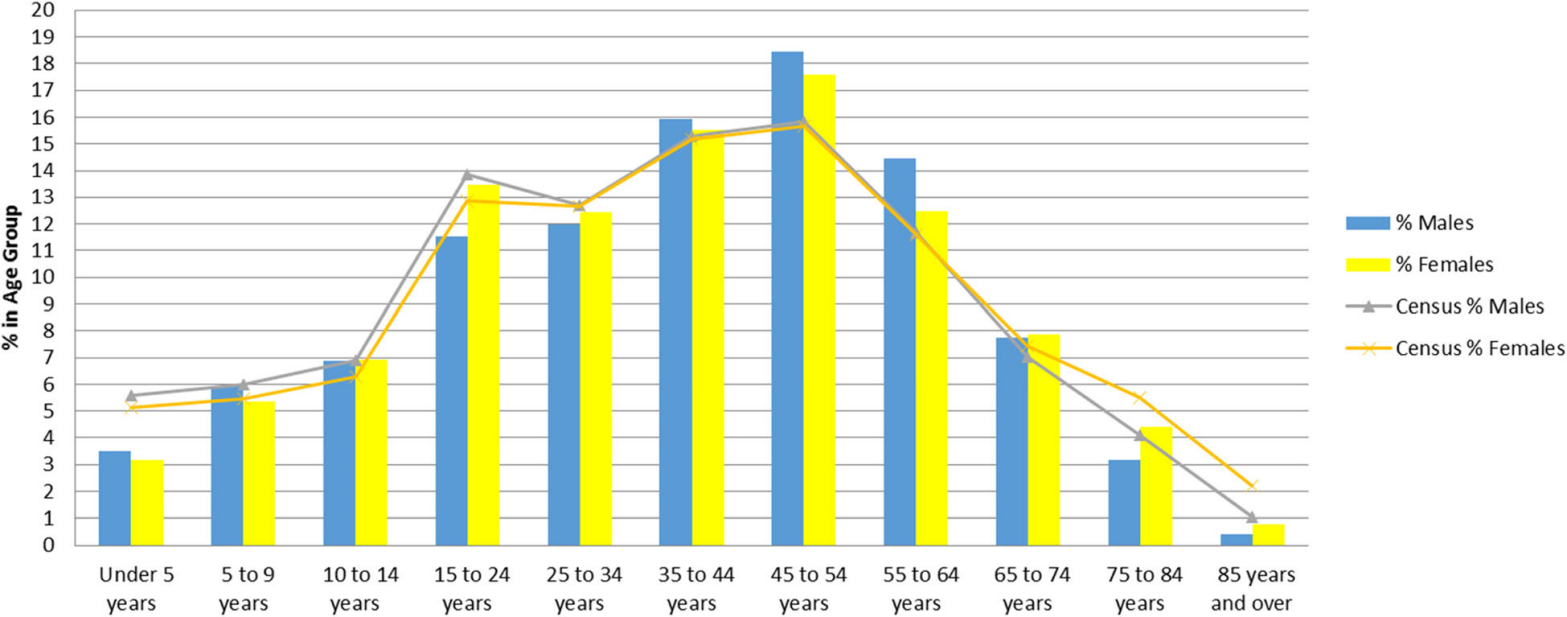
Comparability to the 2006 Canadian Census by Age and Sex – Dataset A

**Fig. 2:**
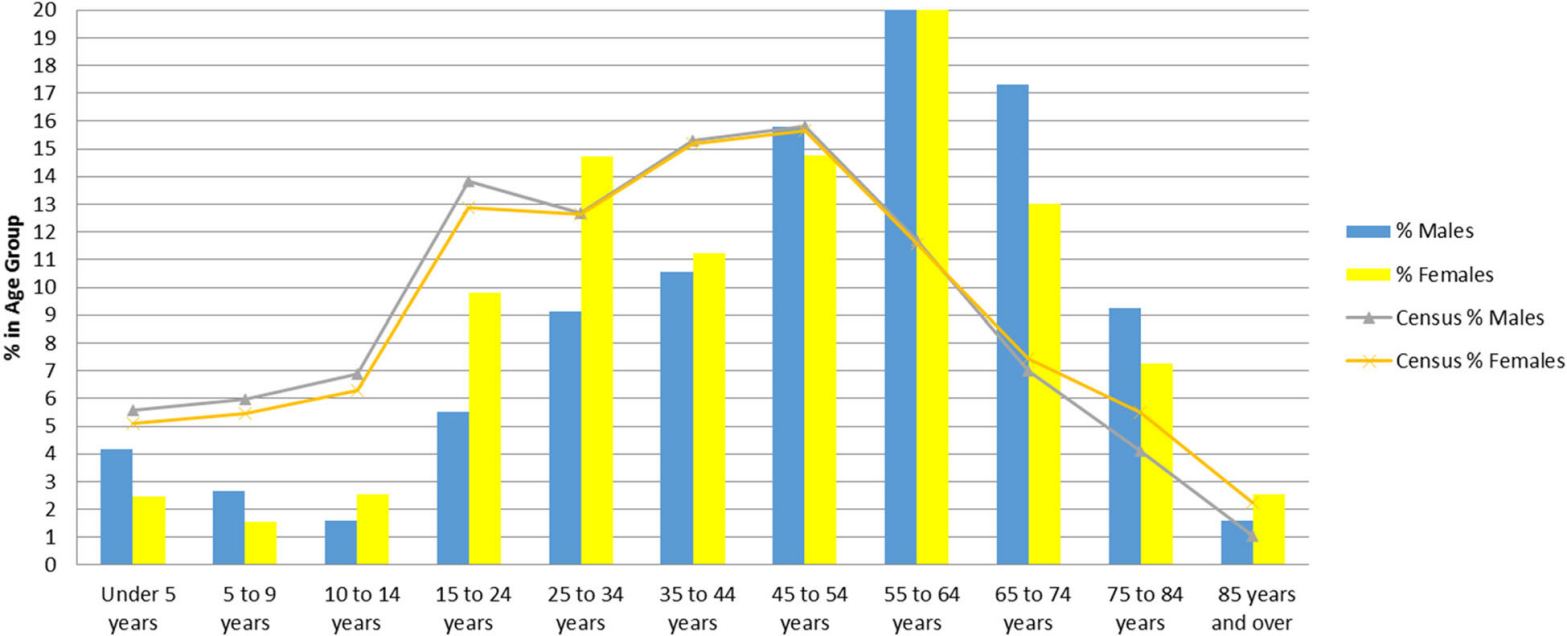
Comparability to the 2006 Canadian Census by Age and Sex – Dataset B

**Fig. 3:**
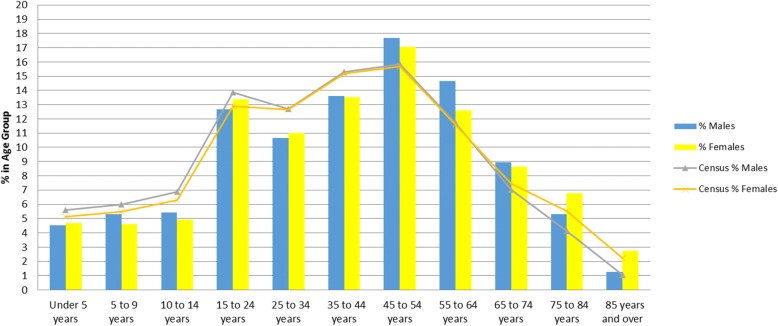
Comparability to the 2006 Canadian Census by Age and Sex – Dataset C

We found that comparability was high among the practice population and the Canadian census population (on age bands and sex) in dataset C, while in dataset A and B significant differences in the population distributions were noted (see Figs.1, 2, 3 and Table 3). The comparability of disease prevalence differed based on condition, for example, the prevalence of diabetes and hypertension was higher than published population prevalence figures, while asthma was lower. Two conditions – hypothyroidism and obesity were comparable.

**Table 3:** Comparability Measures

		Results			
Measure		Dataset A	Dataset B	Dataset C	
Comparison of Database Population with Standard Population	Chi Square by patient age and sex to the 2006 Canadian Census^a^	*p* = < 0.001	*p* = < 0.001	*p* = < 0.001	
					Canadian Census Mean (median) age
	Mean (median) age	39.4 (41.0)	48.7 (53.0)	38.4 (38.0)	(39.5)^b^
		Prevalence, Crude % (Age Standardized %; 95% confidence interval^c^)			Published Prevalence %
Concordance of TestConditions	Diabetes	13.8 (11.3; 11.0–11.7)	12.4 (9.0; 7.9–10.1)	d	6.8^e^
	Hypertension	42.0 (35.5; 35.0–36.0)	23.9 (14.9; 13.5–16.3)	29.8 (20.8; 20.1–21.5)	19.1^f^
	Hypothyroidism	7.5 (6.5; 6.2–6.8)	7.3 (5.9; 5.0–6.8)	5.5 (4.1; 3.8–4.4)	7.1^g^
	Asthma^h^	5.6	*	5.0	21.1^i^
	Obesity	35.2 (36.2; 35.7–36.7)	20.9 (18.7; 17.2–20.2)	24.2 (23.5; 22.8–24.2)	24.1^j^
	Urinary Tract Infection	6.9 (6.6; 6.3–6.9)	*	0.9 (0.8; 0.7–1.0)	k

*cell sizes less than 5 are suppressed

^a^The 2006 Canadian Census was selected because age was measured at the start of each dataset. Two of the three datasets began in 2006

^b^Mean age for Canadian Census is not reported

^c^The 1991 Canadian Census of Population is used as the standard population by Statistics Canada

^d^Diabetes was not measured in Dataset C

^e^Source: Public Health Agency of Canada. 2011. Diabetes in Canada: Facts and Figures from a public health perspective. (https://www.canada.ca/en/public-health/services/chronic-diseases/reports-publications/diabetes/diabetes-canada-facts-figures-a-public-health-perspective.html)

^f^Public Health Agency of Canada Report. 2010. Report from the Canadian Chronic Disease Surveillance System: Hypertension in Canada, 2010. (https://www.canada.ca/en/public-health/services/chronic-diseases/cardiovascular-disease/report-canadian-chronic-disease-surveillance-system-hypertension-canada-2010.html)

^g^Gagnon F, Langlois MF, Michaud I, Gingras S, Duchesen JF, Levesque B. 2006. Spatio-temporal distribution of hypothyroidism in Quebec. Chronic Diseases in Canada; 27 [1]

^h^Asthma prevalence was not age standardized because the definition was limited to patients less than 18 years old

^i^Gershon A.S., Guan J, Wang C, To T. 2010. Trends in Asthma Prevalence and Incidence in Ontario, 1996–2005: A Population Study. Am J Epidemiol 172;728–736

^j^Statistics Canada. 2011. Adult obesity prevalence in Canada and the United States. (http://www.statcan.gc.ca/pub/82-625-x/2011001/article/11411-eng.htm)

^k^Urinary Tract Infections are an acute condition, and do not have a population prevalence for comparison

#### Completeness

Variability in sensitivity values for the test conditions was found, ranging from 12% for obesity in dataset A, to 90% for diabetes in dataset B (see Table 4). For the “consistency of capture” measure, completeness varied from a low of 11% for allergy recording in dataset C, to a high of 83% for medication recording in dataset C. Completeness of blood pressure recording was over 80% in all three datasets, while height ranged from 29% in dataset B to 71% in dataset A, and weight ranged from 60% in dataset B to 78% in dataset A. Significant differences in recording by sex were found for blood pressure, height and weight in datasets A and B, with females having a higher level of recording, while dataset B showed no difference in level of recording by sex. In contrast, significant differences were observed by age group for blood pressure, height and weight recording in all three datasets, with the highest level of recording for patients aged 45–59 years of age. The proportion of patients with diabetes who had a blood pressure recording was high (ranging from 81% in dataset A to 97% in dataset B). For patients taking hypertension medications, completeness of recording of blood pressure was also high - ranging from 76% in dataset A to 100% in dataset B.

**Table 4:** Completeness Measures

		Results (%)		
Measure		Dataset A	Dataset B	Dataset C
Sensitivity	Diabetes	79.2	90.4	^a^
	Hypertension	67.2	60.7	33.8
	Hypothyroidism	49.7	66.7	39.9
	Asthma	0.0	*	58.1
	Obesity	11.5	65.5	14.6
	Urinary Tract Infection	66.7	*	62.9
“Consistency of Capture” [46]	Problem List	71.4	57.1	n/a
	Allergy Record	48.2	46.6	11.0
	Medications	69.8	60.8	83.0
Recording of Blood Pressure, Height, and Weight	Blood Pressure	89.1^b,c^	81.1^c^	87.0^b,c^
	Height	70.7^b,c^	29.3^c^	55.3^b,c^
	Weight	78.3^b,c^	59.5^c^	69.0^b,c^
Recording of Blood Pressure among Patients Requiring a Blood Pressure Measurement	Diabetes Diagnosis	80.7	97.1	^a^
	Hypertension Medication	75.7	100.0	87.7

*cell sizes less than 5 are suppressed

^a^Diabetes was not measured in Dataset C

^b^Significant differences by Sex (*p* < .001)

^c^Significant differences by Age (*p* < .001)

#### Correctness

Positive predictive values were found to be variable for the test conditions and across datasets, ranging from 4% for obesity in dataset B, to 80% for diabetes in dataset A (see Table 5). The presence of a tetanus toxoid conjugate vaccination among those 10 years of age and older was 0% in all three datasets.

**Table 5:** Correctness Measures

		Results (%)		
Measure		Dataset A	Dataset B	Dataset C
Positive Predictive Value	Diabetes	79.5	48.9	^a^
	Hypertension	77.9	58.7	65.8
	Hypothyroidism	60.7	37.6	76.0
	Asthma	0.0	*	16.1
	Obesity	83.5	4.0	61.8
	Urinary Tract Infection	0.1	*	3.5
Unlikely Combinations of Age & Specific Procedures	Tetanus Toxoid Conjugate Vaccination	0.0	0.0	0.0

*cell sizes less than 5 are suppressed

^a^Diabetes was not measured in Dataset C

#### Currency

Recording of weight for patients with obesity within one year of their last visit ranged from 62% in dataset A to 86% in dataset C (see Table 6). Office visits within two months for patients with a positive pregnancy test result ranged from 15% in dataset A, to 63% in dataset C. Blood pressure recording no more than one year prior to a patient’s last visit ranged from 64% in dataset A to 94% in dataset B. Significant differences were observed for males and females in dataset A and C, and by age in all three datasets for blood pressure. For height recording no more than one year prior to a patient’s last visit, values ranged from 30% in dataset A to 42% in dataset C. Significant differences for height by sex were found only for dataset A, however significant differences were found in height recording by age across all three datasets. For weight recording no more than a year prior to a patient’s last visit, values ranged from 45% in dataset A to 62% in dataset B. Significant differences by age were observed for weight recording across all three datasets, while differences by sex were found in dataset A alone.

**Table 6:** Currency Measures

		Results (%)		
Measure		Dataset A	Dataset B	Dataset C
Timeliness of Weight Recording for Patients with Obesity	Weight Recording	62.0	76.4	85.5
Timeliness of Visit for Pregnancy	Visiting Patients	14.7	50.0	62.7
Timeliness of Blood Pressure, Height, and Weight Recording	Blood Pressure	64.4^a,b^	93.7^b^	82.4^a,b^
	Height	30.4^a,b^	33.3^b^	42.4^a,b^
	Weight	45.2^a,b^	61.6^b^	57.5^a,b^

^a^Significant differences by Sex (*p* < .001)

^b^Significant differences by Age (*p* < .001)

## Discussion

In this study we developed eleven measures of primary health care EMR data quality, and tested them within three EMR-derived datasets. We were not able to calculate results for several measures in one dataset because of the way the data were collected in that specific EMR. Overall, we found variability in the results of testing the measures among the test conditions (e.g. sensitivity values were highest for diabetes, and lowest for obesity), among datasets (e.g. recording of height), and by patient age and sex (e.g. recording of blood pressure, height and weight). Several of these results are in keeping with other studies of primary health care EMR data quality in Canada. For example, Singer et al. (2016) found differing levels of the completeness of recording for a set of chronic diseases [15]. The results of this study pertaining to recording of measures such as height and weight differ from Tu et al. (2015), however, overall patterns such as less frequent recording of weight versus blood pressure were similar.

Some of this variability is to be expected. For example, one could anticipate blood pressure would be recorded less frequently among younger age groups. Similarly, the high level of completeness of blood pressure recording among patients with diabetes and those taking hypertension medications is perhaps not surprising. However, other results such as no difference in the completeness of blood pressure, height, and weight recording for male and female patients in dataset B versus datasets A and C, do not have an obvious explanation. Some practice sites may have decided that blood pressure, height, and weight should be universally recorded among males and females. In general, practices may record height less frequently than weight, because height varies less over time than weight. This speaks to the importance of understanding the nature of the data in the context of their potential use. The measures developed for this study help illuminate some of the nuances associated with primary health care EMR data. For example, researchers seeking to answer a question regarding patients with hypertension may want to be aware that these patients could have higher levels of blood pressure recording than other patients, and thus may want to consider a study of medication adherence among these patients as opposed to a study of the prevalence of high blood pressure.

Despite advancement in the field, the most recent primary health care EMR data quality literature focuses mainly on describing process steps regarding the assessment of data quality, or on determining one aspect of data quality such as completeness. Reporting guidelines exist for studies using routinely collected health data [36, 37], which highlight the importance of data quality. However, a small proportion of studies using EMR data report on quality assessments [38], with the exception of studies associated with well-established primary health care EMR databases [39, 40]. This may be partly because there is a lack of consensus on the process steps for assessing data quality, the measures to be used, and finally, what acceptable levels for primary health care EMR data quality are [8]. Creating these standards is a challenging task, given that different data are required for different questions, and the level of quality needed varies with types of data use. Developing and testing measures of primary health care EMR data quality is a necessary foundational step in this task.

Assessing primary health care EMR data quality is a complex process. There are many factors that play into how these data come to be, including: how users interact with the EMR and enter data; the EMR system itself; practice characteristics, such as how external data are incorporated in the EMR [8], and the nature of patient populations [41]. The user of primary health care data needs to be aware of the possible impact of these factors. For example, some software programs provide a cumulative patient profile or “problem list” area of the EMR where current diagnoses can be recorded for a patient in a free text field, while others provide a structured “health condition” section with drop-down lists and coded diagnoses, or both. Thus, even within the datasets in our own database we found we could not calculate all the measures we had developed because of differences in EMR structures. This is a particular difficulty that applies to the Canadian context where a plethora of EMRs are utilized by primary health care practitioners, each with its own configuration [27]. Furthermore, different data extraction tools can produce different results [42], adding an additional layer of complexity to this picture.

While the measures presented here are meant to assess overall EMR data quality, each question that one hopes to answer using EMR data is unique. Therefore, when assessing the “fitness” of the data for its intended purpose [9] one needs to apply both broad considerations captured in the aforementioned frameworks, including the provenance of the data [43], and narrow ones – applying specific quality measures to the data elements that are to be used [8, 37, 44]. If we stay true to a broad conceptualization as fitness for purpose, then each question posed that will be answered through the use of EMR data can be considered unique in the context of data quality. Measures serve as tools that can be deployed in a data quality assessment activity, but they are not sufficient in and of themselves to properly assess data quality in terms of a particular question or project. However, a sustained program of testing measures in a wide variety of jurisdictions, across EMR types – could allow the creation of a standard set of measures of data quality for general use. Over time, these measures could be collected into a library (to be shared widely) which would assist those who seek to conduct and report on their own data quality assessments. We recommend that data users examine the suite of measures available and determine which would be the most applicable in their own particular context as they are conducting data quality assessments. From a broader perspective, guidance also exists in the literature regarding data quality management and the governance of health information [45].

### Strengths and limitations

There are several potential limitations of this study. The first is that our assessment of data quality is focused on the structured data elements within the three EMR datasets – not the narrative or notes portion of the record. This limitation reflects a choice made by DELPHI researchers not to extract the narrative portion of the EMR data, for patient privacy reasons. Based on our understanding of our EMR datasets, the majority of the data needed for the analysis would be found in the structured portion of the EMR data. Second, our assessment of data quality will be generalizable only to three types of Canadian EMR software products. Third, in the Canadian context, diagnostic codes are submitted for billing purposes (used in our case definitions for the test conditions), while in other jurisdictions, diagnoses are not linked to billing. Despite these factors, the three datasets are based on EMR data from a large number of practitioners working within many practice types and communities in Southwestern Ontario. It was not within the scope of this study to systematically assess the individual recording practices among all the DELPHI sites; this would have allowed us more fully explain some of the results. A strength of this study is that it focuses on assessing data quality primarily using data within the EMR itself. This approach is the most feasible method to implement on a wide scale, in contrast to methods using external reference data.

## Conclusion

This paper proposes a basic model for assessing primary health care EMR data quality. We developed and tested multiple measures of data quality, within four domains, in three different EMR-derived primary health care datasets. The results of testing these measures indicated that not all measures could be utilized in all datasets, and illustrated variability in data quality. This is one step forward in creating a standard set of measures of data quality. Nonetheless, each project has unique challenges, and therefore requires its own data quality [46] assessment before proceeding.

## Additional files

Additional file 1: **Appendix A.** - Literature Review - Assessing Electronic Medical Record Data Quality. A brief literature review of approaches to primary health care EMR data quality assessment. (DOCX 37 kb)

Additional file 2: **Appendix B.** - Algorithms to Identify Patients with the Test Conditions. A table outlining the component of the algorithms used to identify patients with the test conditions used in this study. (DOCX 13 kb)

## Abbreviations

DELPHI: Deliver Primary Healthcare Information
EMR: Electronic Medical Record
