# Supplementary material for: A basic model for assessing primary health care electronic medical record data quality
Source: BMC Med Inform Decis Mak. 2019 Feb 12;19:30. doi: 10.1186/s12911-019-0740-0 (PMC6373085; doi:10.1186/s12911-019-0740-0)
Supplement: Supplementary file 1 — Appendix A. - Literature Review - Assessing Electronic Medical Record Data Quality. A brief literature review of approaches to primary health care EMR data quality assessment. (DOCX 37 kb) [file 12911_2019_740_MOESM1_ESM.docx]

Appendix A

*Literature Review - Assessing Electronic Medical Record Data Quality*

Four inter-connected elements make up part of the picture of primary health care EMR data quality assessment: 1) data quality frameworks; 2) dimensions or domains of data quality; 3) data quality measures; and, 4) data quality assessment methods. The EMR data quality literature related to these elements is briefly summarized below.

*Frameworks*

Frameworks are overarching steps or models which serve as guides to data users about what aspects of data quality need to be considered. There are data quality frameworks which are not specific to primary health care ^1-3^ . However, within the primary health care literature, there is existing foundational work^4-7^, as well as two recent frameworks. In the first, Bowen and Lau^8^ created a ten step method for assessing EMR data quality, and a guidebook for data quality assessors^9^ . Second, de Lusignan et al. (2011) defined key concepts which can be used to describe both the quality and origin of primary care data^10^, allowing data users to determine the readiness of the data for research purposes^10^.

*Dimensions or Domains*

Dimensions or domains are conceptual aspects of data quality such as validity and reliability. Though not focused on primary health care, Weiskopf^1^ identified five dimensions of data quality: completeness, correctness, concordance, plausibility, currency^1^. Of these, three (which are consistent William’s original two^11^) were designated as essential: correctness, completeness, and currency^1^. In the Canadian primary health care context, Bowen defined seven dimensions of EMR data quality, which overlap with at least three of Weiskopf’s five dimensions. Foundational work from the U.K. suggested that three dimensions of primary health care EMR data quality are important to address: comparability, completeness and correctness^7^.

*Measures*

Data quality measures may be defined as metrics or indicators of data quality, usually aligned within the domains identified above. Examples include: 1) for comparability, assessing the level of concordance among a practice population’s demographic and disease prevalence profile with that of external populations^7;12^; 2) for completeness, calculation of sensitivity values^13^, and assessment of the recording of clinical measures^14;15^ diseases^16^, documentation of patient visits, referrals and consultation letters^17^; 3) for correctness, the calculation of positive predictive values^13^; and, 4) for currency, assessing the presence of clinical measures within a particular timeframe for patients with specific diseases^7^, and the recording of socio-demographic information^18^ within specific timeframes.

*Methods*

Methods are approaches to implementing data quality measures, summarized by Weiskopf (2013) into seven broad categories: “gold standard, data element agreement, element presence, data source agreement, distribution comparison, validity check, and log review”^1^. More recently, Kahn’s (2016) harmonized data quality framework suggests these methods can be categorized into two contexts – verification and validation^2^. Within verification approaches, data that are available within a system are matched or evaluated against indicators that are internally generated, such as the detection of impossible values for a particular data element^2^. Validation techniques on the other hand, compare internal data values to those found in external sources – often in relation to a benchmark or other reference point ^2^.

Reference List

1. Weiskopf NG, Weng C. Methods and dimensions of electronic health record data quality assessment: enabling reuse for clinical research. J Am Med Inform Assoc 2013;20:144-151.

2. Kahn MG, Callahan TJ, Barnard J et al. A harmonized data quality assessment terminology and framework for the secondary use of electronic health record data. EGEMS (Wash DC ) 2016;4:1244.

3. Canadian Institute for Health Information. The CIHI data quality framework. 2009. https://www.cihi.ca/en/data_quality_framework_2009_en.pdf. Accessed January 19, 2018.

4. de Lusignan S, van Weel C. The use of routinely collected computer data for research in primary care: opportunities and challenges. Fam Pract 2006;23:253-263.

5. de Lusignan S, Hague N, van Vlymen J, Kumarapeli P. Routinely-collected general practice data are complex, but with systematic processing can be used for quality improvement and research. Inform Prim Care 2006;14:59-66.

6. de Lusignan S, Metsemakers JFM, Houwink P, Gunnarsdottir V, van der Lei J. Routinely collected general practice data: goldmines for research? A report of the European Federation for Medical Informatics Primary Care Informatics Working Group (EFMI PCIWG) from MIE2006, Maastricht, The Netherlands. Inform Prim Care 2006;14:203-209.

7. Faulconer ER, de Lusignan S. An eight-step method for assessing diagnostic data quality in practice: chronic obstructive pulmonary disease as an exemplar. Inform Prim Care 2004;12:243-253.

8. Bowen M, Lau F. Defining and Evaluating Electronic Medical Record Data Quality Within the Canadian Context. ElectronicHealthcare 2012;11:e5-e13.

9. Bowen M. EMR data quality evaluation guide. 2012. eHealth Observatory, University of Victoria. http://ehealth.uvic.ca/resources/tools/EMRsystem/2012.04.24-DataQualityEvaluationGuide-v1.0.pdf. Accessed January 19, 2018.

10. de Lusignan S., Liaw ST, Krause P et al. Key concepts to assess the readiness of data for international research: data quality, lineage and provenance, extraction and processing errors, traceability, and curation. Contribution of the IMIA Primary Health Care Informatics Working Group. Yearb Med Inform 2011;6:112-120.

11. Williams JG. Measuring the completeness and currency of codified clinical information. Methods Inf Med 2003;42:482-488.

12. Hassey A, Gerrett D, Wilson A. A survey of validity and utility of electronic patient records in a general practice. BMJ 2001;322:1401-1405.

13. Hogan WR, Wagner MM. Accuracy of data in computer-based patient records. JAMIA 1997;4:342-355.

14. Staff M, Roberts C, March L. The completeness of electronic medical record data for patients with Type 2 Diabetes in primary care and its implications for computer modelling of predicted clinical outcomes. Prim Care Diabetes 2016;10:352-359.

15. Hippisley-Cox J, Hammersley M, Pringle M, Coupland C, Crown N, Wright L. Methodology for assessing the usefulness of general practice data for research in one research network. Health Inform J 2004;10:91-109.

16. Singer A, Yakubovich S, Kroeker AL, Dufault B, Duarte R, Katz A. Data quality of electronic medical records in Manitoba: do problem lists accurately reflect chronic disease billing diagnoses? J Am Med Inform Assoc 2016;23:1107-1112.

17. Tu K, Widdifield J, Young J et al. Are family physicians comprehensively using electronic medical records such that the data can be used for secondary purposes? A Canadian perspective. BMC Med Inform Decis Mak 2015;15:67.

18. Laberge M, Shachak A. Developing a tool to assess the quality of socio-demographic data in community health centres. Appl Clin Inform 2013;4:1-11.
