# Supplementary material for: A basic model for assessing primary health care electronic medical record data quality
Source: BMC Med Inform Decis Mak. 2019 Feb 12;19:30. doi: 10.1186/s12911-019-0740-0 (PMC6373085; doi:10.1186/s12911-019-0740-0)
Supplement: Supplementary file 2 — Appendix B. - Algorithms to Identify Patients with the Test Conditions. A table outlining the component of the algorithms used to identify patients with the test conditions used in this study. (DOCX 13 kb) [file 12911_2019_740_MOESM2_ESM.docx]

Appendix B

Algorithms to Identify Patients with the Test Conditions

| Condition | Definition – Gold Standard | ICD9 Diagnosis Code in Billing Table |
| --- | --- | --- |
| Diabetes Mellitus | Diagnosis on the problem list or insulin prescription, or >=2 oral anti-diabetic agents (excluding Metformin), or 1+ Metformin prescriptions, or >=2 abnormal plasma glucose tests >=3 months apart (fasting plasma glucose>=7.0 mmol/L; casual(random) plasma glucose >=11.1 mmol/L; 2 hour plasma glucose in a 75-g oral glucose tolerance test >=11.1 mmol/L), or HbA1c>=6.5%^1^  Dataset C:  Note – results were not tested for diabetic patients because the dataset does not distinguish between random and casual blood glucose laboratory tests, and the threshold values for the two tests are different. | 250 |
| Hypertension | Patients >=18 years with: (Diagnosis on problem list OR 3+ systolic BP readings above the healthy adult threshold OR 3+ Diastolic BP readings above the health adult threshold - >=140 systolic, or >=90 diastolic) OR (1+ Prescriptions for a diuretic OR 2+ anti-hypertensive oral medications) | 401 |
| Hypothyroidism^2^ | Diagnosis on problem list or Prescription for: Hypothyroidism medications or TSH level>10 | 244 |
| Asthma | Patients <18 years with (Diagnosis on problem list AND 1+ medications) OR (2+ types of medications – a controller and relief medication) | 493 |
| Obesity | Patients >=18 years with: (Diagnosis on the Problem list as obesity OR obese) OR (BMI on Problem List >=30) OR (Calculated BMI 30+ (use height and weight)) | 278 |
| Urinary Tract Infection | Diagnosis on the Problem list AND 1+ oral antibiotic medications | 595 |

^1^ Adapted Gold Standard Definition for Diabetes Case Ascertainment as defined by Harris (2010)

Harris SB, Glazier RH, Tompkins JW, Wilton AS, Chevendra V, Stewart MA et al.: Investigating concordance in diabetes diagnosis between primary care charts (electronic medical records) and health administrative data: a retrospective cohort study. BMC Health Serv Res 2010, 10: 347.

^2^Definition adapted from Hassey (2001). Hassey A, Gerrett D, Wilson A. A survey of validity and utility of electronic patient records in a general practice. BMJ 2001;322:1401-1405.
